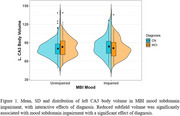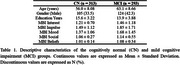# From Structure to Behaviour: Investigating Hippocampal Subfield Atrophy and Behavioural Impairment in MCI

**DOI:** 10.1002/alz70856_100098

**Published:** 2025-12-24

**Authors:** Gursimar Bhalla, Ashwati Vipin, Nagaendran Kandiah

**Affiliations:** ^1^ Dementia Research Centre (Singapore), Lee Kong Chian School of Medicine, Nanyang Technological University, Singapore 308232, Singapore, Singapore, Singapore; ^2^ Lee Kong Chian School of Medicine, Nanyang Technological University, Singapore, Singapore; ^3^ Neuroscience and Mental Health Programme, Lee Kong Chian School of Medicine, Nanyang Technological University, Singapore, Singapore

## Abstract

**Background:**

Mild behavioural impairment (MBI), characterized by the onset of irreversible neuropsychiatric symptoms is the prodromal stage to cognitive decline seen in older adults. MBI may also show comorbid presentation in individuals with mild cognitive impairment (MCI). The relationship between hippocampal subfield volumes and MBI has yet to be established in the context of MCI. This preliminary study thus aimed to identify the bilateral hippocampal subfield volumes directly associated with MBI and to examine the interactive effects of a diagnosis of MCI on these associations.

**Method:**

313 cognitively‐normal (CN) (56.0 ± 8.08 years old; 33.5% male) and 293 MCI (63.1 ± 8.66 years old; 42.3% male) individuals were recruited from the Biomarkers and Cognition Study, Singapore (BIOCIS). Participants underwent 3T T1‐weighted MRI scans and data was processed using Freesurfer software ver 7.2. Participants were administered the Mild Behavioural Impairment Checklist (MBI‐C). Impairment in each of the five subdomains (interest, impulse, mood, social and beliefs) was defined as the presence or absence of at least one symptom within each subdomain. Logistic regression modelling was applied to examine the main and interactive effects of diagnosis and subfield volumes. Age, sex, education years and total intracranial volume were controlled for.

**Result:**

Interest subdomain impairment was associated with lower right parasubiculum volume, with no significant interactive effects of diagnosis. Mood subdomain impairment was associated with lower left parasubiculum volume. The interactive effect of diagnosis however inversed this relationship. Mood subdomain impairment was also associated with lower left CA3 body volume and this effect remained consistent with the interactive effect of diagnosis. Social subdomain impairment was associated with lower left hippocampal tail volume. The interactive effect of diagnosis however inversed this relationship. Beliefs subdomain impairment was associated with higher right hippocampal fissure volume. The interactive effect of diagnosis inversed this relationship.

**Conclusion:**

Overall, atrophy in various hippocampal subfields is associated with the specific, individual MBIC subdomains, and these associations may differ across clinical diagnoses.